# Tutorial on impedance and dielectric spectroscopy for single-cell characterisation on microfluidic platforms: theory, practice, and recent advances

**DOI:** 10.1039/d4lc00882k

**Published:** 2025-02-14

**Authors:** Fatemeh Dadkhah Tehrani, Michael D. O'Toole, David J. Collins

**Affiliations:** a Department of Electrical and Electronic Engineering, The University of Manchester Manchester UK fatemeh.dadkhahtehrani@manchester.ac.uk michael.otoole@manchester.ac.uk; b Department of Biomedical Engineering, The University of Melbourne Melbourne Victoria Australia david.collins@unimelb.edu.au

## Abstract

Cell analysis plays an important role in disease diagnosis. However, many characterisation techniques are labour intensive, expensive and time-consuming. Impedance and dielectric spectroscopy (IDS) offers a new approach by using varying electrical current and electric field propagation responses to probe cell physiology. This review aims to explore the theoretical foundations, practical applications, and advancements in IDS for single-cell analysis, particularly when integrated with microfluidic technologies. It highlights recent developments in electrode configurations, calibration techniques, and data analysis methodologies, emphasising their importance in enhancing sensitivity and selectivity. The review identifies key trends, including the shift towards high-throughput and precise single-cell analysis, and discusses the challenges and potential solutions in this field. The implications of these findings suggest significant near-future advances in biomedical research, diagnostics, and therapeutic monitoring. This paper serves as a comprehensive reference for researchers in different fields to make a bridge between theoretical research and practical implementation in single-cell analysis.

## Abstract 2

Integration of low-frequency electrical impedance and broadband electromagnetic sensing with microfluidic devices enables high-throughput analysis of cell size, membrane properties, and intracellular characteristics.

## Graphical abstract

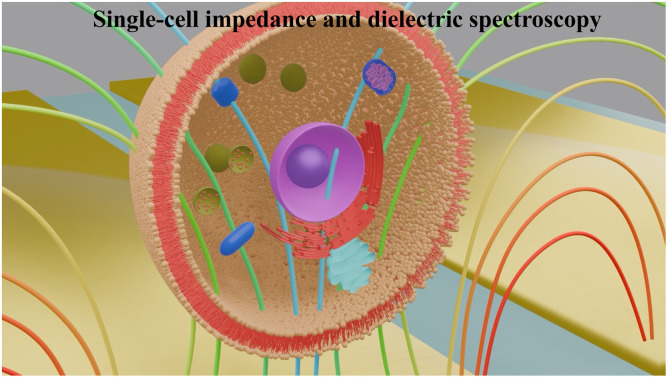

## 1 Introduction

Biological samples collected from patients such as tissue biopsies, bodily fluids, and cells offer valuable insights into various medical conditions. Different aspects of a cell, from its membrane properties^1^ to its DNA sequence,^2^ hold information about its structure and function. These insights find applications in medical diagnosis, therapies, and regenerative medicine.^3^ While previous research often focused on aggregated cell populations,^4^ their complexity and heterogeneity have spurred interest in high-throughput techniques for precise single-cell analysis.

Cell analysis methods include optical, mechanical, chemical, and electrical characterisation. Electrical methods, especially broadband impedance and dielectric spectroscopy (IDS), offer distinct advantages for non-invasive and real-time cell analysis.^5^ There are different terminologies given for techniques used for cell electrical characterisation, which are summarised in Table 1. Here, we define *impedance spectroscopy* as the measurement of a sample's opposition to a varying current at different frequencies, often obtaining bulk properties such as sample conductance and capacitance. The term is commonly used in low-frequency measurement (up to a few MHz) where lumped-element modelling assumptions are valid. *Dielectric spectroscopy* is a form of impedance spectroscopy where material-specific properties are analysed, such as conductivity and permittivity. The term is more common in studies measuring at higher frequencies (a few MHz to GHz) where transmission line assumptions prevail.

**Table 1:** Summary of various terms used for electrical characterisation of cells

Technique	Principle	Applications	Advantages	Limitations	Ref.
Electric IDS	Analysing bulk sample properties in a stationary mode	Tissue analysis, 3D cell culture analysis	Comprehensive spectral information	Low throughput	6, 7
Electric flow IDS	Single cell analysis when cells are passing through a microfluidic channel crossing the sensing zone	Analysis of live/dead cells, identifying cell subpopulations, blood analysis	High throughput and real time	Limited to few frequency points	7, 8
Electric cell substrate IDS	Analysis of cells attached to the surface of electrodes	Investigation of cell adhesion, proliferation, and migration, monitoring the effect of drugs	Sensitive to cell properties, dynamic characterisation of cells	Limited to substrate adherent cells, medium throughput	9
Electrorotation	Analysis of cell rotation when torque is induced by a rotating electric field	Cell membrane and cytoplasm characterisation, cell organelle evaluation	Detailed analysis of cell properties	Precise control of the electric field, time sensitive	10, 11

In cell studies, IDS reveals electrical properties of cell components by exposing cells to electromagnetic fields, which induce changes based on the frequency-dependent behavior of cell membranes, organelles, and structures. IDS can analyse cells without modifications or labelling,^12^ making it suitable for high-throughput analysis and rapid profiling of cell populations down to the single-cell level. Its real-time monitoring capability allows dynamic observation of cell responses to stimuli.^13^ IDS has been widely used in macro-scale systems, analysing blood dielectric properties^14^ and tissue electrical properties to support diagnosis.^15^ Commercial products such as Coulter counters (Beckman Coulter Life Sciences, USA) and impedance-based flow cytometry (*e.g.*, BactoBox®) are employed for cell analysis^16^ and enumeration,^17^ despite calibration challenges. The integration of IDS with microfluidics has broadened its applications by improving analysis precision by reducing sample volume. Miniaturising IDS in microfluidic environments has enabled activities ranging from precise DNA analysis^2^ to advancements in tissue engineering.^3^ While IDS has proven useful for single-cell analysis, its sensitivity to minute changes in cell properties presents challenges. Current efforts aim to enhance sensitivity, selectivity, and cell handling by integrating IDS with sophisticated microfluidic setups, automated systems, advanced data analysis, and specialised electrode designs.

A number of reviews have explored the multifaceted applications of electromagnetic waves for cell characterisation. These reviews span theoretical foundations,^18^ practical applications,^19^ data analysis techniques,^20^ static and dynamic cell analysis,^21,22^ and electrode configurations,^23,24^ as well as challenges in bioimpedance devices,^24^ impedance and microwave sensing methods, position-dependent signals,^25^ cell analysis across the electromagnetic spectrum,^26^ and application of machine learning in impedance data analysis.^27–29^ Given the recent interest in this interdisciplinary field, and its application in various fields of study, this review aims to examine the theory, techniques, designs, and applications of IDS on microfluidic systems, with a special focus on biological and cell studies. In particular, we investigate broadband (kHz–GHz) IDS setups and include insights into calibration techniques, diverse sample handling strategies, and effective data analysis methodologies. Accordingly, this work serves as a reference across fields such as biology, biotechnology, pharmacology and medicine as well as electrical engineering by offering a fundamental understanding of how electromagnetic fields can be leveraged to analyse biological samples at the single-cell level. This accordingly facilitates the advancement of research and practical applications in these domains, bridging the gap between theoretical research and practical implementation.

## 2 Impedance and dielectric spectroscopy theory

**Fig. 1:**
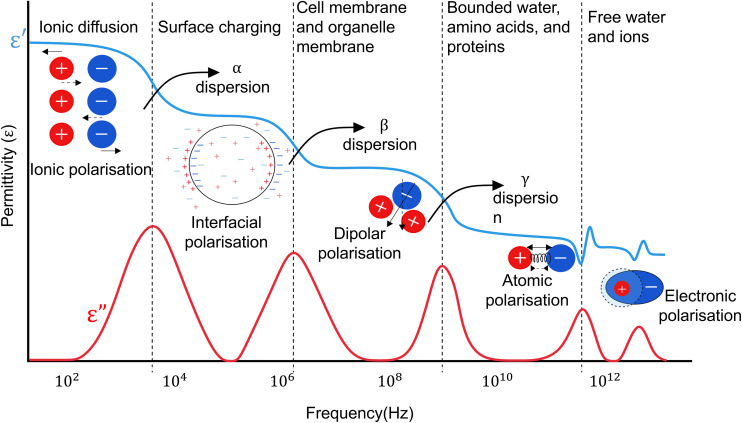
Variation of dielectric *ε*′ and *ε*′′ as a function of frequency and its relevance to biological phenomena in a single cell.

Dielectric materials polarise in a frequency-dependent process when exposed to an electric field. This results in charge displacement, energy dissipation and formation of responses, dictated by the material's composition and structure. These responses, depicted in Fig. 1, include ionic displacement (ionic polarisation), resistance to charge movement across interfaces (interfacial or Maxwell–Wagner polarisation), polar molecule reorientation (dipolar polarisation), atomic stretching (atomic polarisation), and electron displacement (electronic polarisation). This collective frequency dependent behavior of the material forms its dielectric dispersion.

The relaxation frequency of each of these mechanisms is marked by a drop in the real part of permittivity (*ε*′) and a peak in its imaginary part (*ε*′′). For example, at low and medium frequencies (LF–MF), ionic polarisation is dominant. However, at higher frequencies the electric field rate of change becomes faster than the process of ionic polarisation. As a result, the contribution of this phenomenon to the sample's permittivity diminishes and a part of the electric energy dissipates as heat. The frequency at which this shift happens is the relaxation frequency of the polarisation.

**Fig. 2:**
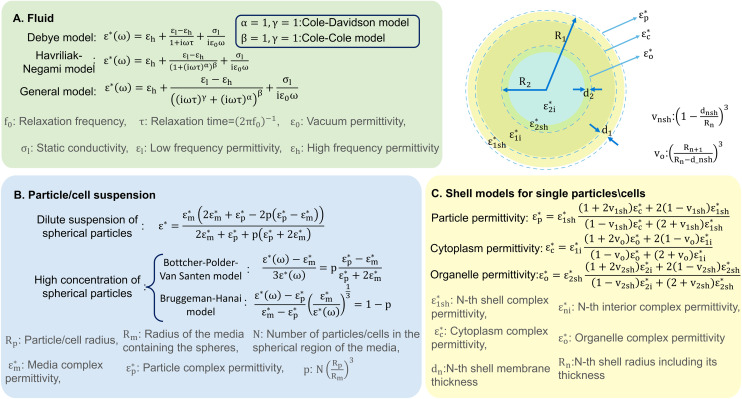
A graphical representation depicting various analytical models developed for analysing A) liquids, B) particle or cell suspensions in liquids, and C) single cells or particles in a liquid.

Dielectric polarisation begins to decay as soon as the electric field is removed, which can be captured by the relaxation function. The relationship between the complex permittivity (ε*(*ω*)) and the relaxation function is established using linear transfer function. For instance, the Debye relaxation function, represented in Fig. 2A, is a specific case of this with the assumption of a single relaxation time (non-interacting dipoles). Addressing the limitations of the Debye model and giving a complete description of dielectric properties, more complex empirical relations have been developed such as the Havriliak–Negami and general models given in Fig. 2A, accommodating conductivity and multiple relaxation times. The general relaxation function of complex systems with a fractal structure with diverse dielectric behaviours can be represented by a constant phase element (CPE).^30^

The dielectric dispersion of a cell over a frequency range of Hz–GHz can be grouped into three regions: *α*, *β*, and *γ*-dispersions, depicted in Fig. 1, with some references defining another dispersion (*δ*) in between *β* and *γ*.^18^ These dispersions provide unique insights into cell properties, as summarised in Table 2. For example, while lower frequencies are usually used to analyse cell size, membrane properties, and quantify cell concentration, higher frequencies can provide information on inner cell properties and organelles, cytoplasm composition, and cellular pathophysiology. Characterising cells using these dispersions necessitates isolating the responses generated solely by the cell, identifying each polarisation mechanism it undergoes, and discerning the cause of impedance changes across different cell types.^30^

**Table 2:** Summary of biological phenomena associated with cell response to electromagnetic fields

Dispersion	Frequency range	Phenomena	Dominant mechanism	Relevance	Ref.
*α*	Hz–kHz	Ionic diffusion at cell membrane, biological processes within cells	Counterion polarisation	Cell presence and enumeration	18, 30
*β*	kHz–MHz	Cell membrane and organelle membrane properties	Interfacial polarisation	Cell shape and membrane integrity	18, 30
*δ*	MHz–GHz	Polarisation of bound water, amino acids, and proteins	Dipolar relaxation	Changes in cell nucleus	9, 18
*γ*	>GHz	Free water polarisation	Atomic polarisation	Cytoplasmic changes	18

This can be accomplished using analytical models originally developed for capacitors with diverse dielectric layers and have been extended to include suspended spheres and colloidal systems, similar to cell suspensions.^31^ Spherical variations of these models are summarised in Fig. 2B.^30^ Wagner's theory provides a model for the complex permittivity of dilute suspensions, while the Bottcher–Polder–Van Santen and Brummagem–Hanai models accommodate higher particle concentrations using effective medium theory. However, these models become complex for single practical analysis, often requiring numerical methods for accuracy.

The shell model,^30^ depicted in Fig. 2C, can be used for single particle/cell analysis. It is based on Maxwell–Wagner theory and represents a particle surrounded by a thin, insulating membrane. The complex permittivity of the system is determined by the permittivity of both the shell and the particle interior. This approach is iterative, allowing for the development of more realistic models of cell structure by adding multiple shells to represent various inner organelles, each enclosed in its own resistive membrane. This theoretical groundwork on the interaction of electromagnetic waves with cells sets the stage for detailed exploration of these techniques' working principles, which we examine in subsequent discussion.

## 3 Applications of impedance and dielectric spectroscopy in on-chip cell characterisation

IDS techniques offer valuable insights into cell size and the electrical properties of its membrane, cytoplasm, and inner organelles. These insights provide an opportunity to assess cell health, viability, differentiation, and behaviours. This review examines their applications in biology and medicine, including tasks such as cell counting, sizing, and discrimination based on size, deformation, and intrinsic properties, along with analysing cell responses to stimuli.

At low frequencies, IDS usually discriminates cells based on their size. Cell size analysis is a common practice in biological studies, though traditional methods such as optical microscopy and imaging flow cytometry^32^ suffer from sensitivity to alignment and experimental conditions.^33^ IDS has been successfully used for: differentiation of live, apoptotic, and necrotic cells,^34^ and circulating tumour cells with larger size and increased membrane capacitance compared to other blood cells;^35^ profiling monocyte and leukocyte activation in type II diabetes, correlating leukocyte size and opacity with cardiovascular risk factors;^36^ demonstrating neutrophil functional traps in glucose-treated neutrophils for diabetes testing;^37^ distinguishing activated T-lymphocytes;^38^ and analysis of the success rate of cell sorting.^39^ Additionally, changes in cell size during mitosis phases have been shown to correlate with impedance signals.^40^ However, current IDS setups lack standardised particles with controlled electrical traits, crucial for benchmarking unknown cells using common techniques such as flow cytometry. This is an active field of research aimed at increasing the application of IDS in cell-based size discrimination.^41^ Furthermore, IDS has recently been employed to optimise the frequency of positive dielectrophoresis based on the cell's biophysical characteristics, improving sorting efficiency of circulating cancer cells.^42^

Moreover, IDS can be used for cell counting as it has been shown that changes in sensing zone capacitance directly correlate with cell quantity.^43^ Recent improvements in integrating neural networks with impedance cytometry have enabled the capture of single-cell signals hidden in the measured signals from cells simultaneously passing through the sensing zone.^44^ This can offer cost-effective alternatives to complex sample preparation steps used in conventional techniques. When IDS is performed at frequencies higher than 1 MHz, cells can be categorised based on their intrinsic (*i.e.* cytoplasm) electrical properties.^45^ Data provided by this analysis can be used to distinguish live and dead cells^46^ with applications in food safety^47^ and medicine.^48^

Building on this, addition of a microfluidic method to induce cell deformation can make this technique applicable in understanding cellular states and diseases which are reflected in their deformability. Common techniques used for cell deformability analysis are atomic force microscopy and micro-pipette aspiration, offering insights into cytoskeletal structure in a low-throughput manner. IDS can provide a high-throughput alternative with the benefit of simultaneous mechanical and electrical analysis of cells.^49^ Cell deformation is induced in these systems by constricted channels^50^ or extensional flow,^51^ permitting contactless operation. Cell deformation can be then translated into size-independent parameters such as cytoplasmic viscosity, capacitance, and membrane tension,^52^ which can then be used to differentiate cell populations.^53^ For instance, specific membrane capacitance and cytoplasmic conductivity can be used to classify sub-types of tumor cells after genotypical or phenotypical modifications.^54^ Information gathered on cell deformation, diameter, and electrical properties has further been used to distinguish leukocyte and granulocyte sub-types.^55^

The application of IDS in classifying cells based on their intrinsic electrical properties, size, shape, and deformability has introduced a new non-invasive approach for studying cellular responses to various stimuli, offering an alternative to conventional methods.^3^ While traditional methods such as fluorescent labelling for cell imaging, flow cytometry, and RNA and DNA sequencing are precise, they often require specialized techniques, compounds, and instruments for sample preparation and analysis. Moreover, their invasive nature renders tested samples unusable for further research. Recently a study^56^ has analysed the effectiveness of measuring electrical deformability of cell shape after mechanically inducing shear stress by comparing the results to optical deformability measurements using a custom made setup, reaching a very high correlation between the two methods.

IDS has been utilised to investigate the effect of chemical^57^ and physical^58^ treatments on cell viability as well as analysis of the reversible electroporation efficiency.^59^ IDS has also been used in analysing the results of antimicrobial susceptibility testing^60^ and analysis of host microbiota susceptibility after antibiotic treatments.^61^ Another study observed changes in RBCs infected by *P. falciparum* over time,^62^ leveraging a multi-shell model to note variations in membrane capacitance, cytoplasmic conductivity, and parasite volume ratio at different infection stages. These findings could aid in distinguishing infected and uninfected cells, potentially benefiting disease diagnostics. While low frequency measurements are sufficient in most of these studies, the cell membrane acts as a barrier, eliminating wave propagation through cell cytoplasm. As a result, most of these studies take into account cell size variations and membrane properties as a feature for differentiation and characterisation of cells.

At higher frequencies, the capacitive barrier produced by the cell membrane can be bypassed, providing insights into the cell's cytoplasm and organelle properties. For instance, analysis of the reflected and transmitted signals enables evaluation of mitochondrial activity^63^ and nucleus size^8^ with applications in cell differentiation based on normal, shrunk, or enlarged nuclei detected through changes in cytoplasm capacitance.^64^ While this evolving field holds promise in understanding cell behaviour and responses to stimuli and can pave the way for more effective diagnostics and targeted therapies, studies in this domain remain limited. The variations in signal amplitude and phase induced by changes in intracellular properties, such as nucleus size, are often subtle, requiring very sensitive setups as well as sophisticated models to establish correlations between these variations and biological characteristics. With the development of advanced techniques with a higher signal-to-noise ratio (SNR) and integration of machine learning techniques, further advancements may be realised. These innovations could enable a more detailed analysis of intracellular properties, including nucleus characteristics, expanding the application of broadband IDS in cellular diagnostics and therapeutics.

Recent advancements in neural networks and deep learning offer a potential contribution to IDS, particularly in rapid cell assessment and classification.^65^ These methods have shown success in low frequency analysis, where impedance data has been used to classify cells based on size-independent electrical properties,^66^ mechano-electrical properties,^52^ and cell deformability^67^ resulting in applications such as five-part leukocyte differentiation.^68^ While the majority of studies integrating machine learning techniques with single-cell IDS have focused on low frequency studies, recent studies have explored higher broadband frequency analysis.

In broadband studies, for example, prediction models have been developed to differentiate the permittivity of two species of viable and nonviable yeast cells at frequencies most sensitive to the difference of live/dead cells.^69^ Moreover, analysis of the entire spectra in the range of MHz–GHz using machine learning algorithms has enabled the evaluation of the chemical treatment effects on cell nucleus size,^64^ and to detect various bacterial species in a high throughput setup.^70^ These advancements highlight the potential of the integration of machine learning with IDS to take advantage of the cellular information captured across a wide frequency range.

In summary, IDS offers a promising alternative to slow, costly, and/or low efficacy techniques that impact samples irreversibly and require large cell quantities. Their integration with optical methods enhances understanding of how cell phenotype correlates with impedance signals.^71^ Some studies propose approaches that merge cell sorting with impedance measurement compartments, creating a sample-in-answer-out platform for blood cell phenotyping.^36,38^ Exploring the optimal frequency to distinguish between cell types is also an intriguing avenue.^72^

## 4 Setup development

### 4.1 Instrumentation

**Fig. 3:**
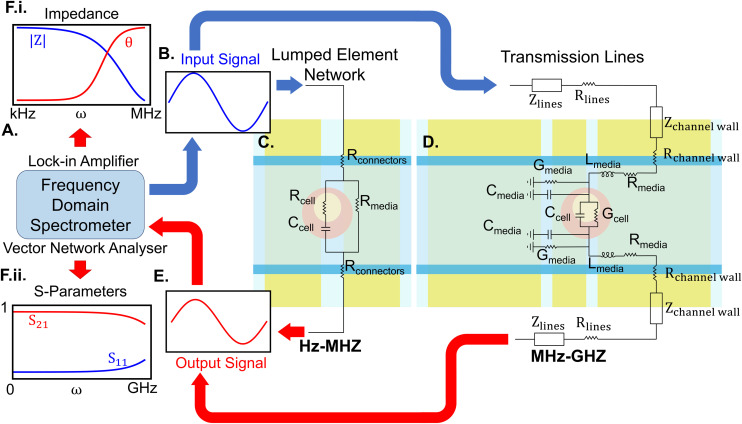
Essential elements of an IDS for single-cell studies. A) A frequency domain spectrometer, which can be a lock-in amplifier or an impedance analyser suitable for low-frequency analysis or a VNA suitable for broadband measurements. B) This instrument inputs a signal with a predefined voltage to the sensor. C) The sensor may be designed for low-frequency analysis, where its components are modelled as lumped elements, D) or for high and broadband frequency analysis, where the electrodes are modelled as transmission lines. E) The output signal is transmitted back to the spectrometer, where the data is represented as either the magnitude and phase of impedance (F.i) for low frequencies, or *S*-parameters (F.ii) for high frequencies.

The essential elements of an IDS for biological studies are frequency domain spectrometers and sensing units which are demonstrated in Fig. 3. The frequency spectrometer outputs an oscillating waveform typically stepping through one frequency at a time and compares this against one or more response waveforms measured synchronously with the output. The spectrometer then returns a demodulated result at each frequency, *i.e.* the phase and amplitude change from output to input or between inputs. Over a bandwidth spanning the RF and microwave regimes, network analysers are utilised, measuring the complex reflection (*S*_22_, *S*_11_) and transmission (*S*_21_, *S*_12_) parameters of a material-under-test (MUT). A scalar network analyser returns the magnitude of these parameters, whereas a vector network analyser (VNA) returns both the magnitude and phase. In studies involving low frequencies (Hz–MHz), an impedance analyser or its equivalent such as a lock-in amplifier applies a sinusoidal potential to the electrodes, measuring the current with respect to the applied voltage. This analysis yields the complex impedance, derived as the ratio between the excitation voltage and the response current. Both these setups should apply a low power to ensure cell integrity.^73^ Recent work has further used on-chip waveform generators and lock-in amplifiers to replace bulk lab instrumentation, enabling the development of wearable and implantable devices.^74^

The sensing unit commonly consists of sets of electrodes positioned around a channel or a cavity containing the MUT. The connection between the frequency spectrometer and the sensing unit requires careful consideration of transmission and parasitic effects, necessitating precise calibration. For example, microwave probes, though preferred for GHz measurement at the micro-scale,^75^ are more prone to parasitic effects and necessitate additional calibration.^76^ Sensing units can either be developed on integrated on-chip setups^77^ or printed circuit boards (PCBs) integrated with microfluidics.^78^ Although cost-effective, PCB-based methods may nevertheless suffer from reduced sensitivity, necessitate additional steps for bio-compatibility and efficient fluid control, and are not transparent. Transparency enables the integration of these setups with microscopes for live measurements.

At frequencies up to a few MHz, electrode structures effectively operate as lumped element networks. However, at higher frequencies where electrical lengths become significant, they can be considered as transmission lines (TLs). At these frequencies, sensors can be broadly categorised into broad and narrow-band. Broadband sensors use planar transmission lines and waveguides, such as microstrip lines, slot lines, and coplanar waveguides (CPW), to observe dielectric changes across a wide range of frequencies. Narrow-band techniques leverage these structures to form resonators to scrutinize dielectric responses at specific frequencies with higher precision.

The performance of broadband platforms is limited by noise from high-loss passive components, reduced SNR near the water relaxation frequency, and lack of a single calibration technique across a wide frequency range (Hz–GHz).^79^ To overcome these challenges, complementary metal-oxide semiconductor (CMOS) technology has been integrated into these setups,^80^ forming interferometric systems,^81^ oscillator sensors,^82^ and micro-electrode arrays with application in high throughput enumeration of cells with a single cell resolution.^74^

By contrast, narrow-band resonators avoid these limitations by focusing on a small number of discrete frequencies. The MUT flowing through a microfluidic channel atop a resonator disturbs the fringing field, altering its capacitance, influencing resonant frequency, and quality (*Q*) factor. Measuring *S* parameters at resonance occurs in a shorter time compared to broad-band measurements.^83^ These changes, translatable through equivalent circuit models (ECMs), reflect the sample's electrical properties within the channel.^84^ For instance, it has been shown that frequency shifts in resonance are proportional to cell volume and inversely related to sensor volume.^26^ However, oscillator stability remains challenging, demanding noise cancellation methods for accurate measurement due to a variable and degrading *Q*-factor.^85^

### 4.2 Design and fabrication

#### 4.2.1 Electrode configurations

**Fig. 4:**
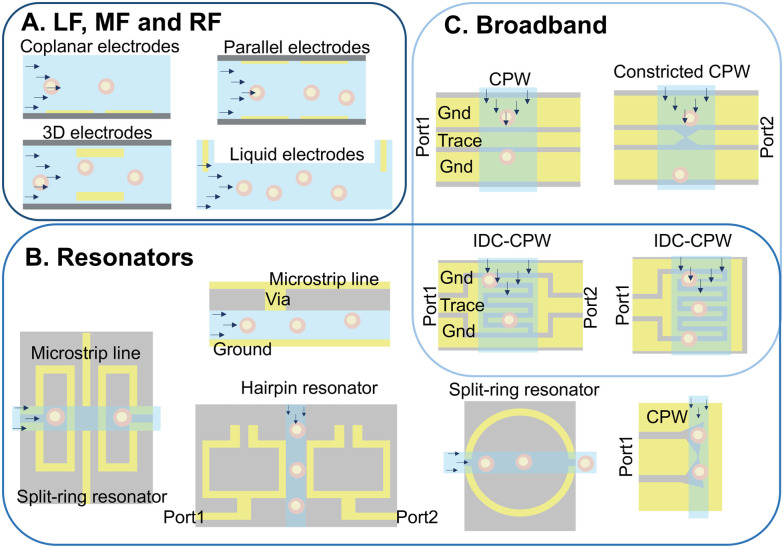
Examples of different electrode configurations used in IDS cell characterisation at low frequency (kHz–MHz) (A), broadband (kHz–GHz) (B) and narrow-band (GHz) (C) setups. Cells can be suspended or cultured in the sensing area, however, here they are flowing through the channels.

Sensor sensitivity and spatial resolution are determined by the electrode configuration. Setups for low-frequency analysis are fundamentally different from those working in higher frequency ranges. In what follows, the electrode configurations most commonly used for each frequency range are covered and summarised in Fig. 4.

##### Low frequency configurations

Common low-frequency electrode designs include coplanar, parallel, liquid electrodes, and micro-electrode array configurations, as depicted in Fig. 4A. Coplanar designs, featuring two or more electrodes in a single plane on one side of the channel, have been extensively employed for cell type analysis,^86^ drug exposure tests,^87^ and mechano-electrical cell property studies through integration with constricted channels^49^ or hydrodynamic pinching.^88^ Despite the ease of fabrication, this configuration produces an inhomogeneous electric field confounding responses with the cell's vertical position as well as electrical properties. Moreover, meaningful data extraction requires extensive post-processing, limiting applicability to unlabeled cell populations.^24^ Increasing the number of electrodes has been shown to enhance information extraction, including particle velocity, electrical diameter, and relative prominence, correlating with particle height from the signal.^89^ Micro-electrode arrays, a subgroup of coplanar electrodes, feature tens to thousands of electrodes on a single surface, each typically analysing a single attached or trapped cell with high precision.^74^

Parallel electrode configurations feature the use of electrodes on the top and bottom of the channel. These have been used for cell differentiation^90^ and tumorigenicity assessment.^12^ Novel configurations have been used to improve sensitivity, including differential measurement setups to reduce background noise^91^ and resonant circuits with series inductors to enhance response current by reducing impedance at resonance frequency.^92^ Despite challenging multi-step fabrication requiring precise alignment, these setups generate more homogeneous electric fields. Solid 3D electrodes, however, offer simpler fabrication, achieved by inserting tungsten needles^93^ into the main microchannel. This comes at the expense of relatively large dimensions, which negatively impact sensitivity for single-cell studies. Enhanced accuracy with uniform electric fields over a small volume can be attained by reducing electrode size with probing gates that are aligned to the flow direction, and can be made with monocrystalline silicon and supported by passive SU-8 pillars.^94^ Facilitating fabrication, heavily doped silicon wafers integrated with readily aligned sidewall micro-electrodes^95^ or silver–PDMS^96^ and carbon–PDMS^97^ setups eliminate sacrificial layer lithography, reducing costs. Alternatively, liquid electrodes utilise conductive liquids to carry current generated at side chambers by coplanar electrodes or prefabricated electrode channels filled with low-melt-point alloys.^98^ This simplifies fabrication and ensures a homogeneous electric field while nullifying the dominating effect of electrode–electrolyte interface capacitance, making this approach suitable for low-frequency and high-throughput cell shape and deformation analysis.^99^

These electrodes are crucial components in commercial Coulter counters, which involve two chambers connected by an orifice and filled with conductive liquids. These systems have been modified into high-throughput on-chip setups,^100^ measuring resistance changes when cells displace the liquid passing through the orifice. However, flow instability affects performance.^24^ Combining liquid electrodes with other configurations such as coplanar^101^ and parallel electrodes^65^ can also be used to compensate for fringing effects with a more homogeneous electric field. Integration with dielectrophoresis (DEP) focusing further enhances signal consistency^99^ and reduces signal dependency on cell position.^66^ DEP focusing exploits electrokinetic phenomena, directing polarisable particles to specific vertical positions in an inhomogeneous electric field. Finally, applying an electric field perpendicular to constricted channels using liquid electrodes enables high-throughput single-cell analysis with reduced current leakage.^66^

##### High frequency configurations

Electrode configurations for resonators and broadband setups are depicted in Fig. 4B and C, respectively. Resonator electrodes are categorised into 3D and planar configurations. 3D electrodes encompass coupled double split-ring and cylindrical dielectric resonators,^83^ substrate-integrated waveguide cavity resonators,^102^ and folded-waveguide cavities.^103^ While 3D electrodes offer improved sensitivity, planar electrodes are more compact, suitable for integrated platforms, and readily compatible with lab-on-chip technologies. However, they may exhibit reduced sensitivity due to lower-quality resonance and smaller fringing electric fields. Efforts are underway to overcome these limitations.^84,104^ Designing resonator electrodes for cell studies with a suitable *Q* factor and sensitivity requires knowledge of the specific frequencies exciting cell properties at the scale of interest.^105^

Planar transmission lines, particularly the coplanar waveguide (CPW) structure, are widely employed for developing broadband sensors, and are effective in cell analysis up to 40 GHz. CPWs, characterised by a central conductor flanked by semi-infinite ground planes, enable quasi-transverse electromagnetic mode propagation with low dispersion. With nearly half of the electrical field concentrated within the fluidic channel and minimal parasitic effects due to smooth transitions between feeding probes and electrodes, the CPW outperforms other planar transmission lines.^106^ The CPW has found application in various cell studies,^107^ with modifications such as capacitive gaps for single-cell analysis.^108^ Moreover, CPW designs with corrugation^109^ and minimized dielectric thickness^110^ can enhance sensitivity. IDCs, despite being resonance structures, can nevertheless be utilised for broadband measurements below their resonance frequency.^111^ Highlighting this flexibility, concentrating the electric field in the microchannel using a CPW increases sensitivity and has been effective in liquid^43^ and cultured cell^111^ analysis from a few kHz to GHz.

#### 4.2.2 Design considerations

The search to enhance sensitivity in measurement setups and detect subtle changes in sample electrical properties remains a continuing area of interest. Studies have highlighted the impact of factors such as channel dimension, electrode configuration, and media conductivity on measured impedance.^21^ A recent study used known shell-covered particles to evaluate the effect of design parameters such as channel height, width and the gap between electrodes on the accuracy of single-cell models showing that channel height has the highest influence, followed by the gap between electrodes.^112^ Moreover, matching electrode size to cell size has emerged as a strategy to boost sensor sensitivity by minimizing the background media's influence on signals.^104^ Increasing voltage can also improve sensitivity, though poses risks such as electrode corrosion, bubble formation, and cell damage.^113^ Most studies accordingly aim for a large sensing volume within a limited voltage range to achieve sensitive cell analysis. Consequently, researchers have explored other methods to address the low sensitivity and improve SNR.

Signal sensitivity is significantly affected by the inhomogeneity of the electric field distribution in the sensing zone, and leads to response dependence on the cell's vertical position.^114^ To enhance device sensitivity, either (1) the electric field homogeneity should be increased, (2) the cell position should be pinpointed in the channel, or (3) the influence of cell position on the response should be eliminated by defining new parameters through post-processing techniques. Modifications such as connecting a via to microstrip stub resonators or relocating the transmission line to the top of the microfluidic channel^115^ can enhance electric field distribution in the sensing zone. Fixing cell positions in the channel can be achieved by reducing the sensing volume, using channels or pores with dimensions on the order of the cell diameter (at the risk of fabrication and clogging challenges), or utilising particle focusing techniques.

#### 4.2.3 Fabrication methods

Reducing the size of the sensing unit to the scale of single cells enhances measurement sensitivity, where microfabrication techniques are ideal for constructing electrodes at this scale. This process involves (1) cleaning the substrate, typically composed of low-loss materials such as quartz, (2) coating with a photoresist layer, (3) pattern transferring *via* a laser-writer or mask aligner, (4) developing the pattern by chemically removing the exposed photoresist, (5) electrode deposition *e.g.*, by electron beam evaporation, and (6) a lift-off process to remove the photoresist layer covered by metal to obtain the final electrode pattern. The microfabrication process has been reviewed previously.^116^

Materials for electrodes (*e.g.*, gold, platinum, or carbon), their size, and the distance between neighbouring electrodes significantly impact sensor performance attributes such as SNR, current density, electric field distribution, and bio-compatibility.^114^ Studies have also underlined the importance of electrode thickness in sensitivity, where increasing electrode thickness to match cell/particle diameters (*e.g.*, 10 μm) enhances capacitive and conductive contrast factors.^117^ Functionalisation or passivation techniques, such as covering electrodes with fibronectin^118^ or parylene C,^119^ respectively, can also be used to enhance biocompatibility.

In low-frequency cell characterisation, electrode polarisation is a major challenge, resulting in charge accumulation on the electrode surface and formation of a double layer with higher capacitance and complex permittivity than the sample.^120^ Methods to mitigate this include using a low-conductivity medium, liquid electrodes, and increasing the electrode surface area (which is inversely related to interfacial impedance and electrolyte resistance). Electrode dimensions have been increased using gold nanostructures,^13^ or forming a porous structure by electrodeposition of platinum black.^121^ However, the latter may lead to uneven electrode polarisability.^86^ Another technique is to reduce interference by shielding the electrodes with materials such as glass covers or thin polydimethylsiloxane (PDMS) layers.^24^

PDMS is often used for channel construction due to its biocompatibility and ease of processing. However, it exhibits high losses at microwave frequencies, though this can be managed by controlling channel thickness.^122^ Replica molding remains popular,^123^ however alternative methods have emerged that mitigate the challenge of electrode alignment, including direct lithography of the microchannel onto electrodes^5^ or SU-8 film lamination^124^ with covers permanently inserted or affixed using mechanical fixtures.^125^ Additive manufacturing could eliminate the need for mold fabrication and post-fabrication cleaning, reducing overall costs. However, limitations in the range of printable materials, printing resolution, and quality necessitate further improvement. For instance, the DragonFly LDM (TM) printer has recently addressed one of these challenges by enabling combined printing of conductive and insulating structures.^126^

### 4.3 Sample handling

**Fig. 5:**
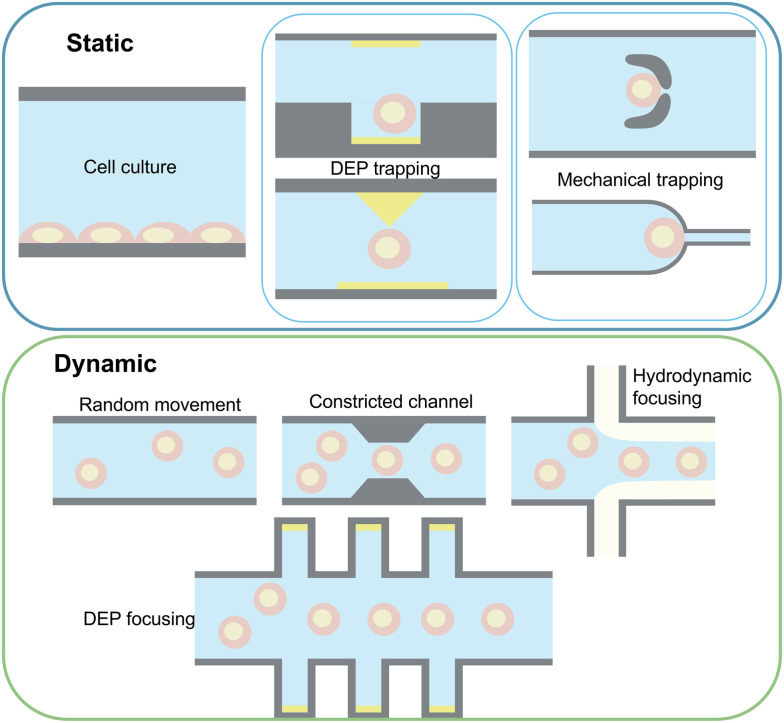
Key sample handling techniques in cell characterisation using IDS.

Measurement setups can be divided into stationary and dynamic categories based on cell movement through the channel, as shown in Fig. 5. Static setups focus on examining cells in a fixed state and typically fall into two groups: trapped-cell and cell-substrate setups. Cell trapping can be accomplished by using dielectric properties of the cells using the DEP technique,^127^ physically trapping cells in micro-structures positioned at the cross-section of the channel width,^108,115^ or using side channels creating cell suction.^128^ Taking advantage of microfluidics, these setups have been used for mechano-electrical characterisation of cells.

While some studies forego trapping techniques,^47^ signal patterns are notably affected by cell positioning, as well as size and intrinsic properties, necessitating reduced cell flow rates for accurate and selective measurements. This is specifically true for broadband measurements where most of the setups use various trapping techniques to enhance SNR. However, these methods are low-throughput, and specific positioning of cells within traps can affect signals, particularly when multiple cells are trapped.^108^

Cell-substrate sensors, feature surface-modified electrode arrays positioned on the channel bottom, facilitating cell adhesion.^129^ Surface modification can be achieved through incubation of sterilised electrodes with cell culture media,^130^ surface activation by a protein promoting cell attachment such as fibronectin^118^ and laminin,^131^ or electrode passivation by adding an insulating layer to avoid contamination and sterilisation.^119^ These devices enable accurate analysis of cell behaviour changes in real-time as cells proliferate, migrate, spread, or detach from the electrode surface.^132^ In such setups, cells shield the electrodes from certain ions in the media,^5^ thereby reducing electrode polarisation. This allows for the investigation of cell properties at low frequencies. These setups are valuable for applications such as drug development,^133^ cancer modelling,^134^ cell differentiation,^135^ 2D^136^ and 3D cell culture.^3^ However, they can be considered unsuitable for measurements at the scale of single cells and require additional cleaning, maintenance, and electrode surface modifications.

In dynamic approaches, cells flow through the electric field concentrated in the sensing zone, allowing high-throughput analysis. Challenges include response dependence on cell position,^90^ which can be mitigated by focusing methods to centre particle stream to the most sensitive area of the sensing zone.^80^ Examples of these techniques are hydrodynamic methods namely, inertial focusing and viscoelastic focusing,^20^ DEP focusing, and constricted channels.^77^ Integration of these approaches for cell focusing can be considered essential in developing sample-in-answer-out platforms^137^ integrated with IDS as demonstrated in ref. 138 where multichannel IDS is integrated with deterministic lateral displacement separation for separation and analysis of heterogeneous samples with wide size distributions. Passive manipulation methods (hydrodynamic focusing and constricted channels) are relatively simple to integrate and have been employed by many studies to enable high throughput IDS by performing pre-positioning of cells relative to electrodes. These techniques have been applied primarily in low frequency studies where 3D hydrodynamic focusing of cells results in increased SNR and enables accurate characterisation of different tumour cells.^139^ Additionally, the addition of constricted channels not only improves cell focusing, but also provides an opportunity to analyse mechano-electrical properties of the cell,^140^ enhancing cell classification and improving the detection success of single-cell IDS. However, this can result in challenges such as channel clogging, depending on the heterogeneity of the input sample. Alternatively, hydrodynamic pinching of cells has emerged as a suitable solution, where the introduction of a sheath flow can both focus cells^141^ and induce deformation, which can be used in a similar manner to a constricted channel, enabling applications such as single-cell mechanical characterisation.^88^

Fluid control traditionally relies on external pressure sources, *e.g.* syringe pumps, balancing controllability and repeatability against device portability. Current explorations aim to relocate liquid control systems onto platforms, recently reviewed by ref. 78. Novel techniques involve particle flow *via* applied static electric fields mimicking DEP particle movement,^142^ eliminating the need for frequent tubing changes, reducing contamination risks and enhancing device portability. However, these methods demand complex fabrication and face challenges in maintaining sample temperature uniformity and consistent repeatability. Moreover, maintaining sample temperature uniformity^143^ is particularly challenging. Temperature variations, particularly notable at higher frequencies where microwave energy absorption elevates sample temperature, must also be controlled to mitigate temperature-induced effects on responses.^107^

Medium conductivity can impact sensor sensitivity, electrode polarisation, and electrolysis. While cells are usually suspended in conductive culture media based on a combination of salts, less conductive liquids such as sucrose can maintain cell viability while reducing these side effects.^46^ However, this can impact cell size and shape over time.^144^ Integration of electrodes with cell purification microfluidics, notably spiral inertial microfluidics^37^ and magnetically activated cell sorting,^145^ allows for the concurrent detection and separation of particles and cells, including circulating tumour cells.^146^ Approaches aiming to induce volume variations among different cells before detection, such as mixing zones in hypertonic solutions, have significantly improved device sensitivity, particularly in live/dead assays.^147^

## 5 Measurement and data extraction techniques

There are several necessary steps to provide data from a sample, namely calibration, signal enhancement, and data analysis. Calibration ensures precision and removes non-sample influences. Signal sensitivity improvement involves employing techniques such as resonance-based methods for high-resolution analysis. Data analysis entails using models such as ECMs to gain insight into cell properties. Each of these aspects is reviewed in this section.

### 5.1 Calibration techniques

Calibration usually involves two steps: 1) establishing reference planes at probe tips and 2) moving the reference planes closer to the MUT by removing the effect of parasitics unassociated with MUT properties, a process known as de-embedding. The first step can be achieved by measuring electrically well-defined standards, including loads, transmission lines (TLs) with various lengths, and resistors. While shorts and opens can be formed by discontinuities in the TLs or a short TL (thru), the load can sometimes be challenging to define. This standard made from an alloy with predefined electrical properties is usually deposited on the wafer, in between ground and trace electrodes. Various on-wafer calibration setups are available, which make use of different compositions of these standards. The choice of method depends on the operational frequency range, with different techniques showing varying error susceptibility.^143^

**Fig. 6:**
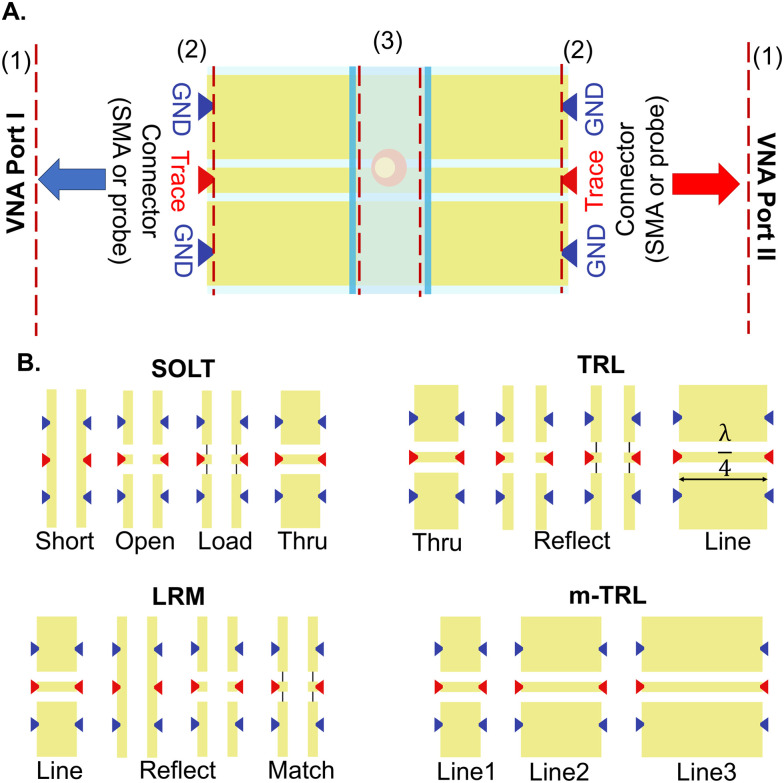
Overview of the calibration technique. A) Two steps of calibration to move reference planes (shown with dashed red lines) from VNA ports (1) to connector tips (2) followed by a de-embedding step to move them further to the sample (3). B) Calibration standards on-chip. Arrows represent the placement of on wafer probes.

As shown in Fig. 6, calibration standards can be defined on the chip and through steps of placing the probes on the standards, measuring *S* parameters, and moving on to the next standard, it's possible to calibrate the VNA to probe tips. The short open load thru (SOLT) method requires these four standards to accomplish calibration in the frequency range of DC to ≈15 GHz. Defining frequency-dependent standards can improve the accuracy of this technique at high frequencies.^148^ Line reflect match (LRM) is based on three standards: a TL as the line, either a short or an open as the reflect, and a load. This method covers a larger frequency range and offers a more compact setup, better suited for automated solutions without the need for operator interference to move the wafer multiple times.^149^ The thru reflect line (TRL) method, which offers better accuracy at higher frequencies, is based on three standards: a short TL as the thru, a reflect (open or short), and a line longer than a quarter wavelength (*λ*) at the centre frequency of calibration.^150^ This method is valid for frequencies where the line length is 20 to 160 degrees longer than the thru line, making this method inapplicable to low frequencies. Based on this, different line lengths should be used for accurate calibration across various frequency ranges. Multiline-TRL (m-TRL) has shown higher precision but requires substantial space, and the wafer probe has to be placed on a high number of lines, increasing the possibility of introducing errors.^151^ However, while other calibration standards (*e.g.*, SOLT, LRM) are commercially available on substrates other than the MUT, TRL and m-TRL can be realized on the same substrate, reducing the errors due to dimension differences.

An alternative technique, single connection calibration, avoids the process of connecting and disconnecting the probes to multiple standards by introducing variations in impedance across the lines using multiple known samples, yielding good agreement with conventional techniques such as m-TRL while minimizing errors over a wide frequency range.^152^ Resources such as the European Metrology Programme for Innovation and Research (EMPIR)^153^ and tools such as StatistiCAL and Matlab Toolboxes such as RF Toolbox streamline the calibration process.^154,155^

### 5.2 Data analysis techniques

The methods utilised for interpreting data obtained from IDS have been reviewed previously for low-frequency measurements.^20^ These techniques mainly differ based on their data analysis. Data extracted from IDS can be analysed directly, focusing on size dependent properties of samples without considering the influence of the measuring techniques or sample conditions. Conversely, data analysis using models involves a de-embedding process to extract an intrinsic and frequency dependent cell property representing the polarisation of the cell and its components in an electromagnetic field. This procedure diminishes non-biological influences through numerical computations, ECMs, finite element models (FEMs), or their combination, albeit at the cost of increased post-processing overhead. Each of these techniques is suited for specific applications, explored further in subsequent sections.

#### 5.2.1 Direct data analysis

This approach involves analysing responses directly, without using models to derive cell intrinsic electrical properties. Over broadband ranges (Hz–GHz), the *S*-parameter magnitude and phase are influenced by variation of the sample impedance, with applications in analysing cell property changes due to chemical treatments^8^ and cancer cell malignancy.^136^ In the lower frequency range, voltage and current measurements are used to determine complex impedance. This has shown use in analysing live/dead cells,^147^ various protozoan pathogens,^156^ cell counting,^84^ and determining osteogenic differentiation and necrosis of cultured cells (both 2D and 3D).^3^ However, as this method relies on cell size, techniques are necessary to minimise size differences within the same cell group or enhance differences between cell groups for better differentiation.

Comparing impedance variations introduced by different cells may not sufficiently distinguish between them, thereby prompting post-processing techniques to define size-independent parameters such as opacity. Opacity, derived from impedance ratios at high and low frequencies,^157^ signifies the electrical transparency of a cell. While valuable for membrane property analysis, at low frequencies (below the relaxation frequency of membrane polarization, about 1 MHz), it primarily reflects cell volume rather than providing effective discrimination.^127^ However, at higher frequencies, cytoplasm conductivity becomes a distinguishing factor. For instance, the ratio of impedance at 19 MHz to 0.5 MHz was found to correlate with acrosome integrity in a fertility study.^158^ Additionally, in GHz measurements, opacity was shown to aid in differentiating cells with similar phenotypes with remarkable sensitivity.^79^

Another metric, phase contrast (*ϕZ*), derived from the variation of impedance phase at high and low frequencies, has shown to be reflective of differences in cell interior conductivity and permittivity. This parameter has shown promise in describing cell electrophysiology and correlating with cell tumorigenicity in certain cancer types.^12^ Moreover, a new metric, the ‘tilt-index’, has been used to extract the exact relaxation frequency of cell membranes from measurements at single or multiple low frequencies. This index quantifies the tilt level of the impedance pulse when a micro-object passes through the electric field.^33^ It has been demonstrated that the tilt level of the impedance signal is affected by intracellular distribution, and increased frequency can elevate the distribution of tilt levels, highlighting the frequency at which the cell membrane becomes conductive.^1^ This index can shift the analysis focus from outer cell properties, such as cell size and membrane electrical properties, to inner characteristics, such as cytoplasm conductivity and organelle properties,^46^ without the need for broadband frequency analysis.

#### 5.2.2 Data analysis using models

The exploration of cell electrical traits involves studying absolute electrical properties such as permittivity and conductivity. These properties cannot be measured directly as they are affected by other electrical elements (*e.g.* media, channel walls, electrode polarisation). Accordingly, modelling techniques such as developing ECM, FEM, or analytical models or using reference materials, can help de-embed these elements from the measurements. Different frequency structures (microwave *versus* lower frequencies) require distinct ECMs (TLs *versus* lumped network elements). At lower frequencies, simpler models involving a resistor (*R*), capacitor (*C*), inductor (*L*), and conductor (*G*) – *RLCG* cell represent the sensor and sample under examination and can be used to extract impedance. Electrode polarisation correction in this method can involve various approaches such as series capacitance,^143^ effective parallel capacitance with conductance,^24^ or a CPE.^5,80^ Microwave frequency TLs are typically characterised by cascades of resistance, inductance, capacitance, and conductance per unit length (PUL). Typically, TLs pass through multiple dielectrics to reach the sample, and calibration lines can be used to define each section by its propagation constant (*γ*) and characteristic impedance (*Z*_c_).

After establishing the sensor ECM, fluidic channel electrical characteristics can be extracted by several techniques. Calibration-free methods are based on the eigenvalue technique where identical TLs with varying lengths (line-line technique) or a single TL covered by different samples (single-line technique) are used to determine the unknown sample's *γ*.^122^ The general line-line approach accommodates material and length variations, proving more suitable for higher frequencies.^159^ Relative measurements can be done using a zero-length channel where a section of the TL is covered by a slab of PDMS resembling microfluidic channel walls without the sample.^109^ Another technique is to use fitting and optimisation methods, such as least squares^125^ or equivalent circuit fitting algorithms^160^ to fit a model to the collected data from a TL covered by the sample. The collected data can be converted into *RLCG* by calculating *γ* from recorded *S*-parameters (after converting them to the transmission matrix).^125^ The model calculates the *S* parameters from an initial guess on *RLCG*. This initial guess can be based on the sample's permittivity and the *RLCG* of the TLs, using developed models and calibration lines to measure the properties of the lines covered by air or channel walls.

While these techniques are based on calculations of *γ*, the reference device technique compares fluid-filled, polystyrene-beads (control particles) suspended in the media, and empty devices to deduce fluid properties without the intricate ECM.^109^ This method is better suited to higher frequencies where the contrast between the *S*-parameters of air and fluid-filled devices is most accurate.^125^ Loading the sensing zone with known fluids is another approach,^161^ enabling permittivity calculation using formulations such as the Havriliak–Negami relaxation.^162^ This technique depends on liquids with known permittivity which is influenced by factors such as temperature. An advancement in this area is based on measurements of reference liquids without presumptions about their permittivities,^163^ yet this also relies on relative measurements, lowering accuracy at lower frequencies.^125^ Using liquids compensates for electrode polarisation by using a reference material with similar conductivity for parameter calculation and subsequent subtraction from sample measurements.^121^

The final step in this process is to relate the measured electrical properties to the sample permittivity. Changes in sample permittivity can be attributed to alterations in either 1) capacitance and conductance or 2) effective permittivity within the sensing area. Techniques such as conformal mapping,^142^ FEM simulations (using software such as Comsol, HFSS, and Q3D),^164^ or direct measurement methods such as the series-resistor technique^165^ can establish the relationship between changes in sample relative permittivity and these factors. By calculating the sensing zone's *γ* or impedance, the sample's permittivity can be extracted. In resonators, this relationship influences the resonant frequency.

**Fig. 7:**
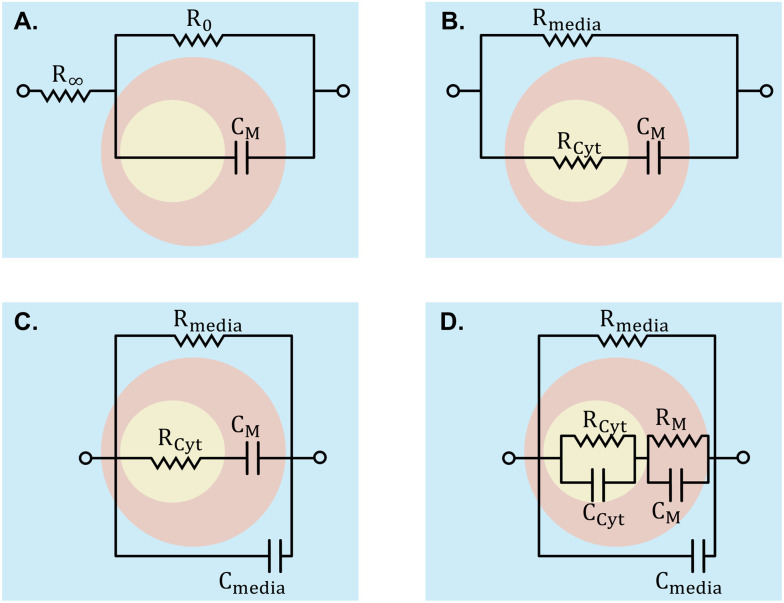
ECMs employed to interpret signals into cell properties. A) Cole model, wherein *R*_inf_ and *R*_0_ denote high and low-frequency resistance, respectively. B) Fricke model. C) and D) are extensions of the Fricke model integrating supplementary elements for increased precision. In these models, while *R* and *C* signify resistance and capacitance, the M subscript designates membrane properties, Cyt represents cytoplasm properties, and ‘media’ denotes properties of the surrounding medium.

To analyse the electrical properties of cells within a sample, various modelling techniques are employed. Creating an ECM using lumped elements is a common method, considering components such as cell membranes, cytoplasm, and extracellular media. These models are depicted in Fig. 7. Models such as the Fricke–Morse circuit assess cell size and polarization, while Cole models examine morphological alterations in cell monolayers concerning *α* and *β* dispersions.^18^ Other models include additional elements to model the cell membrane and cytoplasm more accurately. The fringing field or edge effect, resulting from electric field non-uniformity in the channel, can be corrected by defining a geometrical factor determined by Schwartz–Christoffel mapping.^166^ Numerical methods summarised in Fig. 2, such as single-shell and double-shell models, provide alternatives for cell analysis, enabling investigation into nucleus size effects.^8^

FEM simulation of the sensing zone aids in translating membrane capacitance and cytoplasm resistance to parameters specific to a cell type, thereby enhancing cell differentiation accuracy,^66^ although it requires larger computational resources. Comparative studies of these two methods are available.^166^ For adhesive cells, a statistical technique-based method has been proposed to account for the biological diversity of various cells.^167^ Finally, neural networks have been proposed for converting raw impedance signals to cell properties,^44^ but adequate training is crucial. Developing supervised or unsupervised learning approaches specifically designed for cell impedance spectroscopy has been shown to effectively detect and categorise unknown cells.^44^

#### 5.2.3 Data analysis aided by artificial intelligence

Recent advancements of artificial intelligence (AI), particularly machine learning and neural networks, have made it an asset in single cell IDS, enabling precise and efficient data analysis. As discussed in previous sections, a common technique for analyzing IDS data requires manual data fitting to ECMs or multi-shell models, which is time consuming and requires specialized expertise. With the aid of AI methods, this process can be automated to enable identification of cell properties in real-time. Additionally, they can provide a correlation between the collected data and biological processes, which can provide a better understanding of cell state, activity, and function. As the application of AI in cell impedance spectroscopy has recently been reviewed in more detail elsewhere,^27–29^ we briefly cover the methodology of using AI for single cell IDS here.

Following data acquisition, either in the form of impedance or dielectric properties of the sample, it is necessary to reduce unwanted noises either using filters or by using techniques (covered in the next section) which improve the overall SNR. This step ensures that a clear signal is provided for algorithm training. Consequently, signal features such as time constants, the relationship between amplitude and phase, opacity, tilt index, *etc.* can be used as algorithm inputs. Additionally, normalization or standardization can ensure data comparability and also correct variations due to environmental or instrumental factors. While this process is usually handled manually, it has been demonstrated that the recurrent neural network can also handle raw signal quantification^65^ and classifying them based on signal metrics.^168^

Accordingly, models may either make use of supervised or unsupervised learning. In supervised learning, labels such as cell type,^168^ size and trajectory,^65^ deformability,^98^ state,^169^ or specific physiological condition^170^ are available and the extracted features can be correlated through trained models such as decision trees,^70^ random forests, support vector machines,^171^ or neural networks.^172^ To reduce the impacts of specific conditions in the training data resulting in overfitting the use of multiple convolutional neural networks^44^ and using a parallel setup to measure the training and target data simultaneously for data normalization^170^ have been examined.

On the other hand, algorithms such as *k*-means^173^ or Gaussian mixture models^174^ can group cells based on the similarities of their spectrum, without knowing the number of subpopulations. This yields phenotypes without predefined labels and is useful to identify cells in a heterogenous population.^28^ Further, Honrado *et al.*^169^ combined this technique with supervised training, by feeding the clustered data to a supervised learning method to quantify each subpopulation. Finally, deep learning models, such as convolutional neural networks (CNNs) or recurrent neural networks (RNNs), are especially useful for handling complex and non-linear relationships between spectral features and biological properties. These models have been used to trace cells at multiple channels simultaneously^175^ and classify coincident cells.^44^

#### 5.2.4 Signal to noise ratio improvement

Small sample volumes and potential parasitic couplings, particularly at higher frequencies, pose challenges to achieving high sensitivity. To enhance sensitivity, resonance-based and interferometric techniques are deployed. The latter, known as the differential method, combines signals from reference and test channels. When materials in both channels match, signals cancel destructively removing common confounds affecting both channels (*e.g.* temperature). However, dissimilar materials exhibit relative variation. This method demands precise device symmetry, necessitating specific adjustments such as controlling metal film thickness and automatic channel alignment.^176^ Advancements in miniaturisation allow these setups to target single-cell/particle analysis,^177^ for instance, addition of an interferometric setup has shown to improve the sensitivity of TLs, such as parallel capacitor,^178^ and microstrip lines.^176^ While initially utilised for single frequencies, recent adaptations enable wide bandwidth studies ranging from kHz to GHz, broadening its application scope.^178^

The inhomogeneous electric field in the sensing zone in most setups, especially those based on coplanar and parallel electrodes, makes it necessary to account for the dependence of the recorded signal on the cell's vertical position as a significant source of noise. Fabrication techniques such as particle focusing units can help by aligning the cell on the mid-line. However, post-processing techniques can also be used to quantify and, therefore, compensate for cell position, mostly by assuming that the cell is a spherical particle, at the expense of complex post-processing steps.

Defining new parameters based on signal shape has shown to be effective in considering the effect of cell height. Examples include (1) altering electrode geometry – *e.g.*, star-shaped electrodes^179^ or non-parallel electrodes,^180,181^ (2) analysing peak-to-peak time of current signals (oblique and transverse) and their ratio,^182^ (3) calculating amplitude relative difference and relative prominence using a combination of coplanar and liquid electrodes^101^ or by increasing the number of non-signal electrodes,^182^ or (4) modifying wiring configurations of parallel electrodes, such as applying voltage to diagonally opposite electrodes to extract cell electrical position.^183^ Similarly, digital filters such as the extended Kalman filter^179^ have shown to correct the signal based on particle position. Finally, the Bayesian approach^184^ and neural networks^44^ have shown to be able to decompose the effect of signals of multiple particles passing through the sensing zone into individual particle effects.

## 6 Conclusion and future outlook

Impedance and dielectric spectroscopy shows promise for cell identification and characterisation across fields such as diagnosis, medicine, biotechnology, food, and liquid analysis. This review offers a guide for implementing IDS for cell analysis and characterisation. Key steps involve selecting relevant frequency ranges, setting up the appropriate measurement configuration, employing calibration, and processing signals to extract information. This review accordingly offers a comprehensive reference of current technological advancements.

Despite progress, several areas require further attention. Low-frequency analysis remains prevalent for applications such as cytometry, membrane analysis, and cell response to stimulation. However, electrode polarisation effects at these frequencies present challenges for sensitive measurement. Recent advancements using neural networks show promise in identifying cell viability or infection status. While high frequencies can be used to interrogate specific cell features, GHz resonators have limited bandwidths, restricting their ability to simultaneously detect large-scale features. Enhancing the resonator *Q* factor for characterising cells in media with similar properties is also essential. Conversely, broadband analysis holds theoretical promise for comprehensive insights into cell properties, but faces challenges in sensitivity, impedance matching, and throughput. Advancements in cell trapping and analysis speed could alleviate these limitations.

The future of on-chip broadband IDS promises versatility across a range of applications. Optimising electrode configurations, cell handling techniques, and integrating machine learning for real-time data analysis can further improve this technique. Machine learning algorithms enable prediction of cell condition as well as classification of cells in real-time. This technique enables simultaneous analysis of various parameters such as membrane resistance, cell electrical radius, *etc.*, from a single dataset, accelerating research in drug screening and diagnostics with a high sensitivity. Exploring molecular–cellular interactions at microwave and millimetre-wave frequencies could also offer insights into their effects on cell behaviour.^119^ Further research may focus on signal sensitivity to cell properties and enhancing sensor sensitivity across high and low frequencies, potentially through tailored electrode configurations. Transition of these setups to point-of-care applications requires miniaturized wave form generators, reducing the need for expensive equipment and simplifying calibration processes for non-specialists. Recent advancements in VNA-on-chip, lock-in-amplifier-on-chip, and waveform generator-on-chip hold promise for research translation to clinical applications.

## Data availability

This review does not include any primary research results, software, or code. No new data were generated or analysed as part of this work.

## Conflicts of interest

The authors have no conflicts of interest to declare. All co-authors have seen and agreed with the contents of the manuscript and there is no financial interest to report. We certify that the submission is original work and is not under review at any other publication.
